# CytoSorb® Hemoadsorption as a Promising Tool to Handle COVID-19-Induced Cytokine Storm

**DOI:** 10.1155/2021/9937499

**Published:** 2021-10-12

**Authors:** Alice-Christin Acevedo, Michael Zoller, Christina Scharf, Uwe Liebchen, Michael Irlbeck, Ines Schroeder

**Affiliations:** Department of Anesthesiology, LMU Hospital, Ludwig Maximilians University, Munich, Germany

## Abstract

Accumulating evidence suggests that a patient subgroup with severe COVID-19 develops a cytokine release syndrome leading to capillary leakage and organ injury. Recent publications addressing therapy of cytokine storms recommended new extracorporeal therapies such as hemoadsorption. This case report describes a 59-year-old SARS-CoV-2-positive patient with severe ARDS. Due to severe hyperinflammation with concomitant hemodynamic instability and progressive renal failure, combination of continuous renal replacement and CytoSorb® hemoadsorption therapy was initiated. Treatment resulted immediately in a control of the hyperinflammatory response. Simultaneously, lung function continued to improve accompanied by profound hemodynamic stabilization. We report the successful utilization of CytoSorb® hemoadsorption in the treatment of a patient with SARS-CoV-2-induced cytokine storm syndrome.

## 1. Background

Accumulating evidence suggests that a subgroup of patients with severe COVID-19 suffers from a cytokine storm syndrome. The cytokine storm is a life-threatening systemic inflammatory syndrome involving elevated levels of circulating cytokines and immune cell hyperactivation that can be triggered by various conditions [1]. It is characterized by capillary leak syndrome and hemodynamic instability, leads to organ injury (e.g., kidney injury (8%), acute cardiac injury (22%), and arrhythmias (44%)), and is associated with a high mortality [2–4].

Given this severe immune dysregulation with a resulting generalized hyperinflammatory response, Ronco et al. recommend considering new extracorporeal support therapies, such as hemoadsorption devices specifically designed to remove cytokines and other circulating mediators [5]. CytoSorb® hemoadsorption represents a promising therapeutic tool in the management of the cytokine storm caused by the hyperinflammation in critically ill COVID-19 patients. The active matrix consists of highly biocompatible, porous polymer beads capable of extracting middle molecules up to a size of 55 kDa from blood mainly via size exclusion and hydrophobic interactions. With a total surface of >45,000 square meters, the adsorber might be able to effectively lower high cytokine levels over time [6]. CytoSorb® has been primarily developed and marketed for cytokine adsorption in the treatment of septic shock and other inflammatory syndromes such as hyperinflammation postendocarditis and pancreatitis in adult patients. There are several studies showing the benefit of CytoSorb® in patients with septic shock and other hyperinflammatory syndromes [7–10]. Recently, Schultz et al. demonstrated that the use of CytoSorb® can reduce mortality in patients with septic shock [11]. However, it has also been shown to reduce endogenous and exogenous compounds from blood such as myoglobin, bilirubin, and others resulting in the CE marking for treatment of associated diseases with increased plasma levels of these substances. We report the successful utilization of CytoSorb® hemoadsorption in the treatment of a 59-year-old patient with SARS-CoV-2-induced cytokine storm syndrome.

## 2. Case Presentation

A 59-year-old male patient (90 kg, 180 cm) with a history of chronic lymphocytic leukemia (CLL) was admitted to an external hospital with fever and cough on March 18, 2020. An external PCR test was positive for SARS-CoV-2 infection. Subsequently, on March 19, 2020, he was transferred to the intensive care unit (ICU) of our university hospital due to acute respiratory insufficiency leading to an endotracheal intubation and progressive deterioration of his general condition (SAPS II 77, SOFA 15). Based on the Berlin criteria, a severe ARDS was diagnosed [12]. The severity of illness was reevaluated by SAPS II and SOFA score every 24 hours. SARS-CoV-2 infection was confirmed by another PCR test in our hospital two days after admission.

The following day and despite positive fluid balancing and broad anti-infective therapy including meropenem, vancomycin, azithromycine, itraconazole, and acyclovir, an increase in interleukin 6 (IL-6) plasma level (4118 pg/mL) was noticed accompanied by fever and hemodynamic instability with norepinephrine demand of 1.2 mg/h.

**Figure 1:**
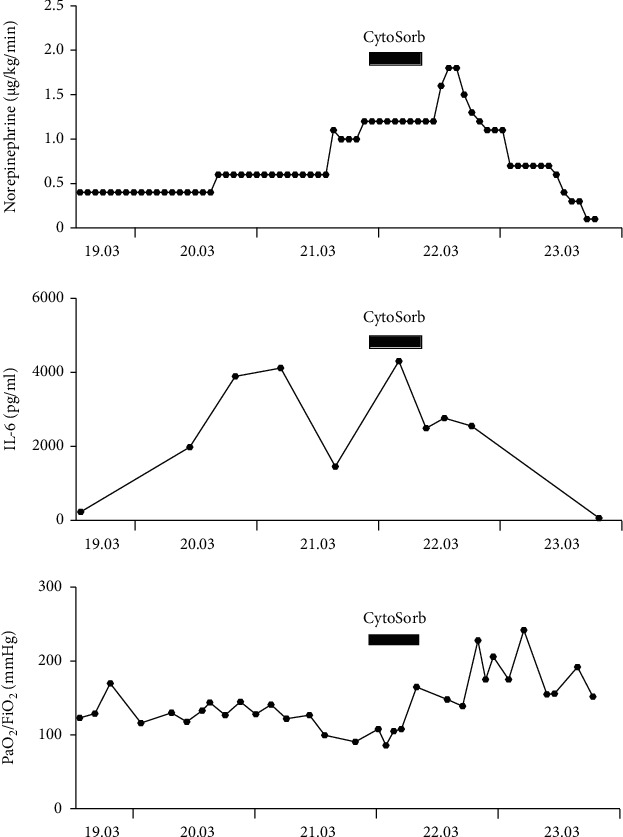
Course of norepinephrine, IL-6, and PaO_2_/FiO_2_ pre-, during, and post-CytoSorb treatment.

In addition to broad anti-infective therapy, intermittent prone positioning was initiated. In the evening of March 21, 2020, the patient developed a generalized hyperinflammatory condition with markedly elevated inflammatory parameters (IL-6 4302 pg/mL) and acute renal failure (KDIGO 3), requiring initiation of continuous renal replacement therapy (CRRT). The patient was treated with CiCa ®-CVVHD (MultiFiltrate, Fresenius AG, Germany, citrate anticoagulation). CRRT (using a high-flow dialysis catheter) was operated with a blood flow rate of 100 mL/min, dialysate flow of 2000 mL/h, ultrafiltration of 250 mL/h, citrate flow of 4 mmol/L, and calcium flow of 1.7 mmol/L. We additionally administered intravenous unfractionated heparin with a target partial thromboplastin time of 40-50 seconds in the presence of leukostasis in underlying CLL and for thromboprophylaxis in SARS-CoV-2 infection. At this point, after escalated anti-infective therapy failed to decrease inflammatory markers and a further clinical deterioration, we installed a CytoSorb® hemoadsorber into the CRRT circuit in order to control the hyperinflammatory response. The cartridge was placed before the hemofilter. Within 30 minutes after the initiation of CytoSorb® treatment, IL-6 levels had already dropped to 2495 pg/mL, and within 24 hours, hemodynamic stabilization began while norepinephrine requirements could be progressively reduced. Simultaneously, PaO_2_/FiO_2_ increased from 90 to 165 mmHg under Bilevel Positive Airway Pressure (BIPAP) ventilation (SAPS II 70, SOFA 15) (Figure 1). CytoSorb® therapy was terminated after 9.2 hours of uninterrupted application in the morning of March 22, 2020. After termination of CytoSorb® therapy, hydrocortisone treatment was started for supportive therapy. During the next two days following discontinuation of CytoSorb® treatment, hyperinflammation could be kept well under control with IL-6 levels dropping further to 20.8 pg/mL (SAPS II 68, SOFA 12). Negative fluid balancing was possible, and norepinephrine administration was tapered off three days after cessation of hemoadsorption therapy. Analgosedation could be reduced, and the patient could be progressively weaned from ventilation. Ten days after CytoSorb® discontinuation, the patient was extubated (SAPS II 68, SOFA 8). Over the next days, he remained cardiopulmonary stable and could be finally transferred to the normal ward 18 days after admission (SAPS II 38, SOFA 1).

## 3. Discussion and Conclusions

This case report describes the successful use of CytoSorb® hemoadsorption treatment as an important part of a multimodal treatment approach in the intensive care context to face a cytokine storm of a COVID-19-positive patient.

The cytokine storm is characterized by a massive release of proinflammatory cytokines with IL-6 playing a key role in its pathophysiology [13]. Currently, there is no evidence-based therapy for the cytokine storm induced by COVID-19, but different treatment approaches such as tocilizumab (TCZ) have been used in the present pandemic analogous to the treatment of cytokine storms of other etiologies. The cytokine absorber CytoSorb® has so far been used primarily for the therapy in septic shock [7, 8] or systemic hyperinflammation of noninfectious origin [9, 10]. The procedure has also shown good results in the treatment of the cytokine storm conditioned by a CAR-T cell therapy [14, 15]. Extracorporeal cytokine removal proved to significantly reduce IL-6, IFN- *γ*, TNF-*α*, IL-1*α*, and IL-1*β* [14, 15]. Due to a similar clinical picture and the suspected similar pathomechanism of the cytokine storm after CART-T cell therapy, CytoSorb® hemoadsorption was used in our department for the treatment of the presented COVID-19 patient with severe ARDS. Compared to TCZ, CytoSorb® can on the one hand eliminate cytokines other than IL-6 and has on the other hand fewer side effects. This could be an advantage compared to the therapy with TCZ.

After 9.2 hours, CytoSorb® treatment was terminated. It has been shown that IL-6 elimination slows down during a 6-hour treatment period, possibly due to the limited capacity of the device or due to decreasing circulating concentrations over time. Based on these data, we decided to use the device for an application period of <12 hours [16]. Following CytoSorb® treatment, a rapid hemodynamic stabilization was observed accompanied by discontinuation of norepinephrine therapy and a relevant improvement in pulmonary function. CytoSorb® therapy in combination with further supportive measures such as hydrocortisone therapy (100 mg Bolus; 10 mg/h continuously) and protective ventilation strategies might have supported the observed clinical improvement. Hydrocortisone is regularly used as a supportive treatment for therapy-refractory septic shock, although its benefits have been widely debated recently [17]. Furthermore, an improvement in pulmonary function as well as in hemodynamic stabilization in the context of CytoSorb® application has already been described in several case series [15, 18, 19]. Dexamethasone was not administered at that time, as the patient was treated prior to the publication of the impressive results of the RECOVERY trial [20].

We assume that the reduction in cytokine plasma concentrations by CytoSorb® was a necessary condition for the following hemodynamic stabilization of the patient. In addition, the CytoSorb® therapy can be terminated at any time and does not lead to any side effects, whereas glucocorticoid therapy, in contrast, can lead to the well-known side and long-lasting effects.

There is currently no targeted therapeutic option for critically ill intensive care patients with a cytokine storm in SARS-CoV-2, as the clinical picture is still not completely understood [21]. A larger number of studies investigating CytoSorb® as a promising tool to handle COVID-19-induced cytokine storm are therefore urgently needed. Therapy with CytoSorb® within the framework of a multimodal therapeutic concept has nevertheless been included in various international guidelines [22, 23]. The application of CytoSorb® in this case was feasible and safe with no adverse or any device-related side effects documented during or after the treatment.

According to current knowledge, CytoSorb® does not cause any significant undesired elimination of physiological blood components such as albumin, platelets, and protein levels beyond what can be expected within the scope of general hemofiltration or dialysis. Since the coagulation and complement systems are not activated by the surface of the substrate, there is little loss of platelets [24, 25]. In our case, there was no significant decrease in albumin level or platelet count after the application of CytoSorb® (Table 1). A potential removal of drugs, for example antibiotics, should be monitored closely, and drug doses should be adjusted if necessary [26]. Therapeutic drug monitoring has not yet become routinely established during the use of CytoSorb®.

We are convinced that CytoSorb® treatment showed a clear benefit within the framework of a multimodal therapy concept through hemodynamic and respiratory stabilization. Despite severe ARDS, a good outcome could be achieved for this patient.

## Figures and Tables

**Table 1:** Laboratory results pre-, during, and post-CytoSorb® treatment. Initiation of CytoSorb® therapy from 03/21/2021 (09:45 pm) to 03/22/2021 (07:00 am).

	WBC (G/L)	Platelets (G/L)	Lactate (mmol/L)	Albumin (g/dL)	Hb (g/dL)	IL-6 (pg/mL)
03/19/2020 07:00 am	306	190	0.8	2.9	9.6	230
03/20/2020 07:00 am	297	224	1.0	2.7	9.2	1983
03/21/2020 07:00 am	309	273	0.8	2.4	9.2	1458
03/21/2020 07:00 pm	306	273	1.0	2.4	8.9	4302
03/22/2020 07:00 am	325	270	1.8	2.3	9.7	2554
03/23/2020 07:00 am	298	273	1.2	2.3	8.1	66.3
03/24/2020 07:00 am	261	270	1.3	2.1	7.3	20

## Data Availability

The data used to support the findings of this study are included within the article. The datasets used and/or analyzed during the current study are available from the corresponding author on reasonable request.

## Abbreviations

ARDS: Acute respiratory distress syndrome
BIPAP: Bilevel positive airway pressure
CAR-T: Chimeric antigen receptor T cells
CLL: Chronic lymphocytic leukemia
COVID-19: Coronavirus disease 2019
CRRT: Continuous renal replacement therapy
CVVHD: Veno-venous hemodialysis
IL-6: Interleukin 6
KDIGO: Kidney Disease: Improving Global Outcome
SAPS: Simplified Acute Physiology Score
SARS-CoV-2: Severe acute respiratory syndrome coronavirus 2
SOFA: Sequential Organ Failure Assessment.
